# Dynamic Processes and Mechanical Properties of Lipid–Nanoparticle Mixtures

**DOI:** 10.3390/polym15081828

**Published:** 2023-04-09

**Authors:** Fan Pan, Lingling Sun, Shiben Li

**Affiliations:** 1 School of Data Science and Artificial Intelligence, Wenzhou University of Technology, Wenzhou 325035, China; 2 Department of Physics, Wenzhou University, Wenzhou 325035, China

**Keywords:** dynamic process, mechanical property, lipid–nanoparticle mixture, dissipative particle dynamic simulation

## Abstract

In this study, we investigate the dynamic processes and mechanical properties of lipid nanoparticle mixtures in a melt via dissipation particle dynamic simulation. By investigating the distribution of nanoparticles in lamellar and hexagonal lipid matrices in equilibrium state and dynamic processes, we observe that the morphology of such composites depends not only on the geometric features of the lipid matrix but also on the concentration of nanoparticles. The dynamic processes are also demonstrated by calculating the average radius of gyration, which indicates the isotropic conformation of lipid molecules in the *x*–*y* plane and that the lipid chains are stretched in the z direction with the addition of nanoparticles. Meanwhile, we predict the mechanical properties of lipid–nanoparticle mixtures in lamellar structures by analyzing the interfacial tensions. Results show that the interfacial tension decreased with the increase in nanoparticle concentration. These results provide molecular-level information for the rational and a priori design of new lipid nanocomposites with ad hoc tailored properties.

## 1. Introduction

With the development of nanotechnology, nanoparticles of various shapes and sizes have been extensively used in biomedical, liquid sensing, fuel cell, packaging and other fields because they can impart different properties such as mechanical, optical, thermal and rheological properties of materials [1,2,3,4,5,6]. However, the effects of nanoparticles on material properties are varied, and these potential applications require the efficient control of the distribution of nanoparticles in the matrix. Amphiphilic molecules such as lipids and block copolymers, which possess both hydrophilic and hydrophobic moieties, can self-assemble into a variety of periodic microstructures (spheres, cylinders and lamellae), making them ideal scaffolds for organizing nanoparticles into well-defined nanostructures, such as nanoplates, nanowires or nanospheres [2,5,7,8,9,10,11,12]. Due to the easy preparation of block copolymers, the phase behavior of nanoparticles in block copolymers has been studied extensively, both experimentally [13,14] and theoretically [7,8,9,15].

In contrast to block copolymers, lipid molecules, which are one of the major constituents of biological systems, as well as various commercial products, such as foods and cosmetics, are basic components in nature. More than 3,000 unique lipid structures have been reported according to the Lipid Maps Structure Database (LMSD). Lipid molecules commonly contain one or more polar hydrophilic functional groups and one or more flexible hydrophobic fatty acid chains [11,16,17,18]. Given the amphipathicity of head and tail chains, lipids can self-assemble into many different structures, either in aqueous solutions or in melts [11,12,16,18,19,20,21]. Phospholipids, usually with one head hydrophilic group and two tail hydrophobic chains, are the most familiar lipid structures, since they are the main components of biofilms and often exist in the liquid environment. Other types of common lipids that are composed of one hydrophilic group and one hydrophobic chain, such as monoelaidin (ME), mono-olein (MO), monovaccenin (MV) and monolinolein (ML), can self-assemble into a series of bicontinuous cubic structures in the water environment or form crystalline phases, usually of lamellar structures and with zero or low hydration, which are quite different from phospholipids [16,20]. A very accurate phase diagram of glycerol a mono-oleate/water system was reported by Qiu et al. based on X-ray diffraction measurements, which was described in the water concentration range from the dry state to full hydration and in the temperature range from −15 to 55 ${}^{{}^{\circ}}$C. It shows that with no water or in the presence of a small amount of water, glycerol mono-oleate forms a lamellar crystal phase or a liquid crystal phase at temperatures under 37 ${}^{{}^{\circ}}$C, while with water content of 20–40%, it forms a gyroid cubic crystalline phase and that with the addition of more water to a concentration of more than 40%, a diamond cubic liquid crystalline phase is formed [20]. The liquid crystalline phases of these polar lipids have high solubilization capacity for lipophilic, hydrophilic and amphiphilic guest molecules and can protect molecules against oxidation or hydrolysis. Thus, they are expected to have more applications than polymers and have received sufficient research interest with respect to their applications in various pharmaceutical, food and biotechnical areas, such as for use as a drug delivery matrix for peptides, proteins, vitamins and amino acids [12,16,21,22,23].

The full use of these liquid crystal phases in such applications calls for understanding of their structures and mechanical properties at the molecular level. Computer simulations offer a unique approach to explore the microstructures and have a certain predictability. For example, self-consistent field theory (SCFT), which is the most widely used method to estimate phases, is usually adopted to predict the phase of polymers and the distribution of nanoparticles in the polymer matrix [24,25,26]. However, SCFT can only observe the probability distribution of configurations in a potential field because it is a method based on phase-field theories; it cannot capture polymer dynamics and the packing structure of polymer chains in detail. Compared to SCFT, molecular dynamics (MD), which simulate the motion of molecular systems following Newton’s equations with an initial configuration and velocity, can resolve the limitations of SCFT [27,28,29,30]. However, the system size and the time scale of classical MD are not sufficient for investigating large-scale systems. To overcome the shortcomings of both SCFT and MD, dissipative particle dynamics (DPD) based on a coarse-grained (CG) model was developed to investigate the equilibrium or nonequilibrium properties of such systems. DPD is the inheritance and development of MD and LGA(lattice-gas automata) simulation; it can obtain the spatial position and velocity distribution information of each particle in the time evolution process just like MD. It also eliminates the concept of lattice, which greatly reduces the systematic error and allows a large time scale to cause the system to be in equilibrium with a limited computational load. Due to these advantages, DPD has been applied to a number of applications and been proven to reproduce the expected behavior successfully, such as in the study of polymer solutions [31], block-copolymer–nanoparticle composites [32] and lipid bilayer membranes [33]. For example, Cai et al. studied the self-assembly behavior of poly(γ-benzyl-L-glutamate)-block-poly (ethylene glycol) (PBLG-b-PEG) block copolymer blended with gold nanoparticles both by experiment and by DPD simulation and showed that the PBLG-b-PEG block copolymer self-assembled into cylindrical micelles in pure form. However, when introducing gold nanoparticles, the formed aggregate morphology transformed from long, cylindrical micelles to spherical micelles. Moreover, the nanoparticles were mostly found near the core/shell interface and in the core center of the micelles. Their DPD simulation results were found to be in good agreement with their experimental observations [34]. Kranenburg and Smit performed DPD simulations on a mesoscopic model of a phospholipid molecular structure containing a head group of three hydrophilic beads and two tail chains varying in length from four to seven beads. They investigated the phase behavior of double-tail lipids with varied tail length, headgroup interactions and temperature, which can reproduce the experimentally observed phases [35]. However, to the best of our knowledge, DPD simulation of lipid–nanoparticle mixtures is limited. Hence, we can address the issue by systematically modeling equilibrium structures of a binary mixture of lipid molecules and nanoparticles in a melt by using a DPD simulation based on a coarse-grained (CG) model. In this work, we select lipid molecules with one head chain and one tail chain to investigate the phase behavior of lipid–nanoparticle mixtures under different concentrations of nanoparticles. We are also interested in the dynamic processes and mechanical properties of lipid–nanoparticle mixtures.

## 2. Method and Model

### 2.1. Method

The simulation was based on the DPD method, which was originally developed to simulate hydrodynamic interactions during molecular dynamic simulation [36,37,38,39,40]. In the DPD method, three categories of forces were introduced to describe the movement of beads, in accordance with the Newton’s law, where conservation force ($F_{ij}^{C}$) represents the force required to exclude the volume effect, dissipative fore ($F_{ij}^{D}$) represents the viscous resistance among moving beads and a random force ($F_{ij}^{R}$) typifies a stochastic force. These forces occur between a pair of beads: the *i*-th and *j*-th beads. Thus, the total force on the *i*-th bead can be expressed as follows:

(1) $$F_{i}=\sum_{i\neq j}\left(F_{ij}^{C}+F_{ij}^{D}+F_{ij}^{R}\right).$$

The conservative force is the soft repulsion acting along the intermolecular vector, which is presented as follows:

(2) $$F_{ij}^{C}=a_{ij}w\left(r_{ij}\right){\hat{r}}_{ij},$$

where $a_{ij}$ indicates the maximum repulsive force between the *i*-th and *j*-th beads, and $r_{ij}=\left|r_{i}-r_{j}\right|$, ${\hat{r}}_{ij}=\left(r_{i}-r_{j}\right)/r_{ij}$. The weight function ($w\left(r_{ij}\right)$) can be expressed as follows:

(3) $$w\left(r_{ij}\right)=\left\{\begin{array}{cc} 1-\frac{r_{ij}}{r_{c}} & r_{ij}<r_{c}\\ 0 & r_{ij}>r_{c} \end{array}\right.,$$

where $r_{c}$ is the cutoff radius. The dissipative force is a hydrodynamic drag force, which is presented as follows:

(4) $$F_{ij}^{D}=-\gamma w^{2}\left(r_{ij}\right)\left({\hat{r}}_{ij}\cdot v_{ij}\right){\hat{r}}_{ij}.$$

Here, $v_{ij}=v_{i}-v_{j}$. The random force corresponds to the thermal noise, which is represented as follows:

(5) $$F_{ij}^{R}=\sigma w\left(r_{ij}\right)\zeta_{ij}\Delta t^{-1/2}{\hat{r}}_{ij},$$

γ is the friction coefficient, and σ is the noise amplitude. γ and σ are related as $\sigma^{2}=2\gamma k_{B}T$, where *T* is the absolute temperature, and $k_{B}$ is the Boltzmann constant. $\zeta_{ij}$ denotes a random number from a uniform random distribution with unit variance and Gaussian distribution. σ=3.0 and γ=4.5 are usually used as standard values in the simulation.

### 2.2. Model

**Figure 1:**
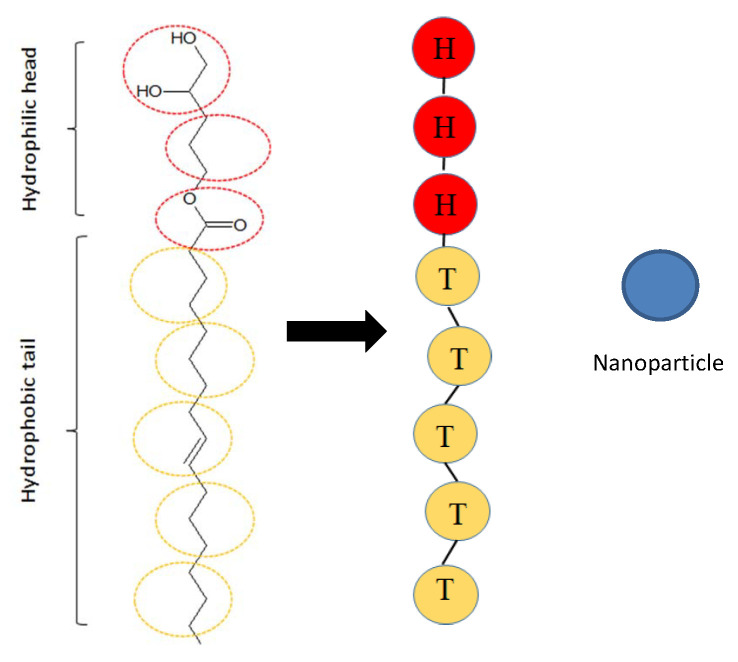
DPD model of the lipid molecule and the nanoparticle. The atomistic representation of a lipid molecular structure consisting of one head group and one tail group is demonstrated in the leftmost column, and the corresponding coarse-grained model is shown in the middle column. Hydrophilic head beads are indicated in red, and hydrophobic tail beads are represented in yellow. The rightmost side shows a schematic representation of the nanoparticle, which is indicated by blue color.

Our simulation is based on a coarse-grained (CG) model that treats a small group of atoms as a single bead located at the center of mass of the group. The atomistic lipid corresponding to our simulation model is glycerol mono-oleate(1-(cis-9-Octadecenoyl)-rac-glycerol), which contains a hydrocarbon chain, an ester bond and a glycerol backbone. The two remaining carbons of the glycerol moiety are free and confer polar characteristics to this part of the molecule. Thus, this part is hydrophilic and we commonly called it the head chain. In contrast, the C18 hydrocarbon chain, which formed a cis double bond at the 9,10 positions, is strongly hydrophobic, and we commonly referred this part as the tail chain. In our work, we adopt a single type of coarse grain to model head-chain or tail-chain beads according to their amphipathicity of atom groups, which is similar to the work of other researchers [35,41]. The mapping of atomistic lipids and the corresponding model used in this paper is shown in Figure 1, where the hydrophilic beads (H) and hydrophobic tails (T) are shown as red and yellow beads, respectively. Two neighboring beads in one lipid chain are connected by a harmonic spring force with spring constant $k_{s}$ and equilibrium bond length $r_{s}$:

(6) $$F_{ij}=k_{s}\left(1-\frac{r_{ij}}{r_{s}}\right){\hat{r}}_{ij}.$$

Here, we set the parameters $k_{s}=120.0$ and $r_{s}=0.7r_{c}$ to make the bonded chain structure, which are similar to previous worcks [42,43,44]. In this paper, the rigidity of the head particles in the lipid structure is greater than that of the tail particles; thus, an additional angular harmonic potential energy based on angular potential constant ($k_{\theta }$) and equilibrated angle ($\theta_{0}$) is set to make a rod-like chain structure. The angular harmonic bending force is presented as follows:

(7) $$F^{\theta }=-\nabla \left[k_{\theta }{\left(\theta -\theta_{0}\right)}^{2}\right],$$

where θ is the angle between two adjacent bonds from the center particle (i). We set $k_{\theta }=6.0$ and $\theta_{0}=\pi$ for three consecutive particles to ensure a stiff rod chain with enough mobility for self-assembly. In addition, the nanoparticles were modeled as a single bead and shown as blue beads in Figure 1.

There are also several works providing more accurate coarse-grained models. For example, Shelley et al. described a method for developing a CG model for phospholipids by fitting some potential parameters on the basis of comparisons, which can semiquantitatively reproduce the density profile of an aqueous dimyristoylphosphatidylcholine (DMPC) bilayer [45]. Marrink et al. optimized the current CG models in terms of four aspects: speed, accuracy, applicability and versatility. Their CG model was proven to be versatile in studying nonlamellar systems [46]. Compared to their models, it seems that our approach is less refined. Nevertheless, the coarse-grained models we cite here are qualitative rather than quantitative in their predictions, so they still have certain validity.

### 2.3. Parameters

In the DPD method, reduced units were usually used for convenience. The cut-off radius ($r_{c}$) represents units of simulated length; bead mass (*m*) defines units of simulated mass, and $k_{B}T$ defines the unit of simulated energy. In addition, the time was scaled as normalized units (τ). The cutoff radius can be estimated as $r_{c}$ = ${\left(\rho V_{b}\right)}^{1/3}$, where $V_{b}$ and ρ represent the volume and particle density of a DPD bead, respectively. In our simulation, the modified version of the velocity–Verlet algorithm proposed by Groot and Warren was used to integrate the motion process [47]:

(8) $$\begin{array}{c} r_{i}\left(t+\Delta t\right)=r_{i}\left(t\right)+\Delta tv_{i}\left(t\right)+\frac{1}{2}{\left(\Delta t\right)}^{2}f_{i}\left(t\right)\\ {\tilde{v}}_{i}\left(t+\Delta t\right)=v_{i}\left(t\right)+\lambda \Delta tf_{i}\left(t\right)\\ f_{i}\left(t+\Delta t\right)=f_{i}\left(r\left(t+\Delta t\right),\tilde{v}\left(t+\Delta t\right)\right)\\ v_{i}\left(t+\Delta t\right)=v_{i}\left(t\right)+\frac{1}{2}\Delta t\left(f_{i}\left(t\right)+f_{i}\left(t+\Delta t\right)\right). \end{array}$$

We selected the time step Δt=0.01τ, where the time unit (τ) is defined as follows [48]:

(9) $$\tau =\sqrt{mr_{c}^{2}/k_{B}T}.$$

We set repulsive interaction parameters as $a_{ii}=25$ for the same types of particles and as $a_{ij}=100$ for different types of particles in the simulation, which were applied extensively in previous works [47,49,50]. The Flory–Huggins parameter (χ) can be calculated from the relationship between $a_{ii}$ and $a_{ij}$, that is, $\chi =0.286\left(a_{ij}-a_{ii}\right)$. This formula has been adopted in previous simulations [51]. We listed the interaction parameters between different DPD beads in Table 1. All simulations were performed in a cubic box with a volume of V=L×L×L under periodic boundary conditions. We performed the calculations for box sizes ranging from $L=25r_{c}$ to $L=35r_{c}$ to avoid the finite size effect [52]. Then, we optimized the size of the simulation box as $L=30r_{c}$. The whole system was implemented with the NVT ensemble using a large-scale atomic/molecular massively parallel simulator (LAMMPS).

**Figure 2:**
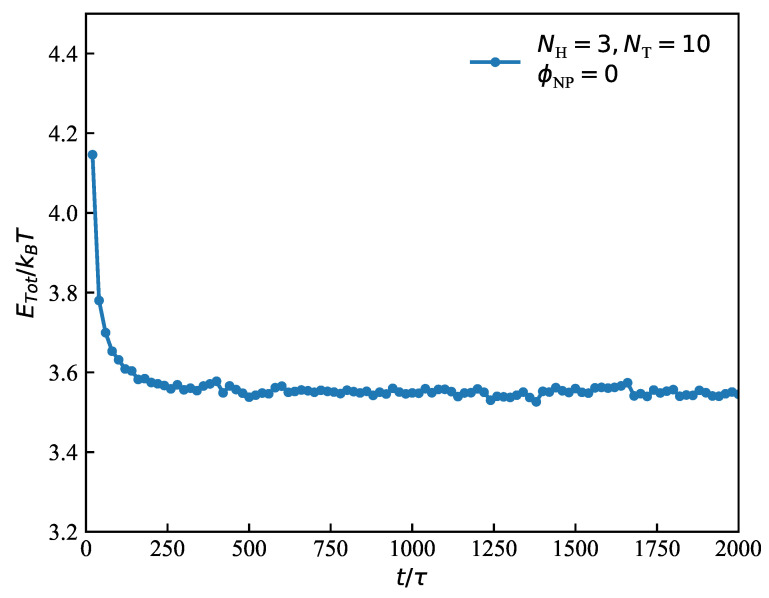
An example of obtaining the equilibrium state in the dynamic process with parameters $N_{H}=3$, $N_{T}=10$, the total energy $E_{\mathrm{Tot}}/k_{B}T$ as a function of time steps.

In general, the equilibrium state can be achieved after about 200,000 DPD time steps in the dynamic process. In this state, the energy decreased to the lowest value. Taking the system with pure melt lipid molecules as an example (Figure 2), we observed that the energy of the microstructure eventually decreased to a gentle state. Since the initial structures have a strong influence on the final results of simulations, we usually input several different initial structures to compare the energy of their stable structures, such as the preassembled lamellar and hexagonal structures, as well as random inputting, and we selected the equilibrium state with the lowest energy in all studied systems. After determining the initial structure of the lipid matrix, we randomly placed nanoparticles in the initial structures. In our present work, we performed about 300,000 DPD time steps and 6 rounds of DPD simulations with different random seeds in all simulations to ensure the acquisition of equilibrium structures and the repeatability of the findings. The physical quantities are presented as the six averaged parallel simulations dates Section 3 to minimize error and obtain a better representation.

## 3. Results and Discussion

**Figure 3:**
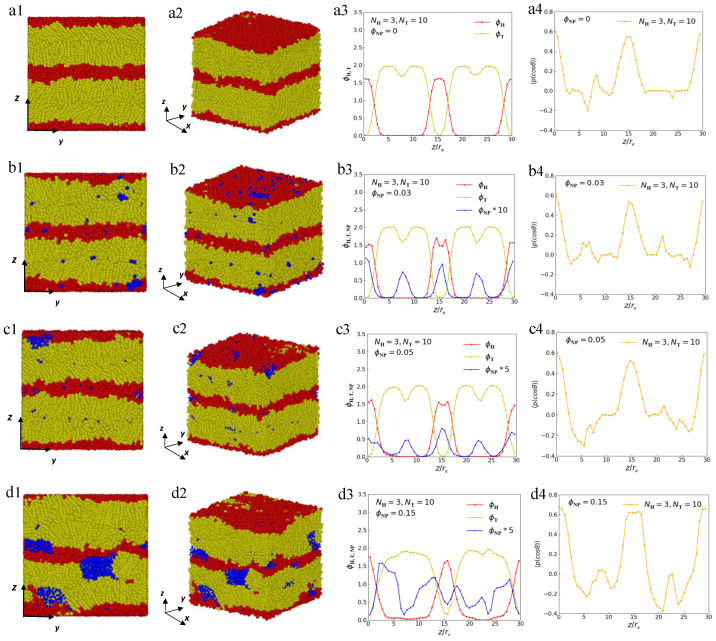
Representative lamellar microstructures of lipid–nanoparticle mixtures with $N_{H}=3$ and $N_{T}=10$. The microstructures for different views, density profiles and order parameter profiles are listed on the left side, middle and rightmost side, respectively. (**a1**) The front view of pure lipid molecule with $\phi_{\mathrm{NP}}=0$. (**a2**) The side view of pure lipid molecule. (**a3**) The density distribution of pure lipid molecule along the *z* direction. (**a4**) Order parameter profile of pure lipid molecule. (**b1**) The front view of lipid–nanoparticle mixtures with $\phi_{\mathrm{NP}}=0.03$. (**b2**) The side view of lipid–nanoparticle mixtures with $\phi_{\mathrm{NP}}=0.03$. (**b3**) The density distribution of lipid–nanoparticle mixtures with $\phi_{\mathrm{NP}}=0.03$ along the *z* direction. (**b4**) Order parameter profile of lipid–nanoparticle mixtures with $\phi_{\mathrm{NP}}=0.03$. (**c1**) The front view of lipid–nanoparticle mixtures with $\phi_{\mathrm{NP}}=0.05$. (**c2**) The side view of lipid–nanoparticle mixtures with $\phi_{\mathrm{NP}}=0.05$. (**c3**) The density distribution of lipid–nanoparticle mixtures with $\phi_{\mathrm{NP}}=0.05$ along the *z* direction. (**c4**) Order parameter profile of lipid–nanoparticle mixtures with $\phi_{\mathrm{NP}}=0.05$. (**d1**) The front view of lipid–nanoparticle mixtures with $\phi_{\mathrm{NP}}=0.15$. (**d2**) The side view of lipid–nanoparticle mixtures with $\phi_{\mathrm{NP}}=0.15$. (**d3**) The density distribution of lipid–nanoparticle mixtures with $\phi_{\mathrm{NP}}=0.15$ along the *z* direction. (**d4**) Order parameter profile of lipid–nanoparticle mixtures with $\phi_{\mathrm{NP}}=0.15$.

**Figure 4:**
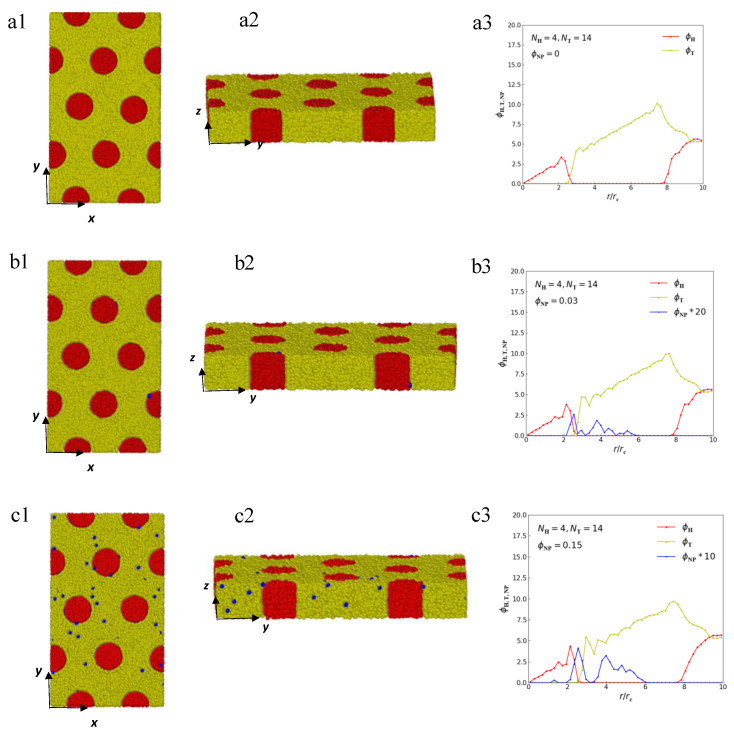
Representative hexagonal microstructures of lipid–nanoparticle mixtures with $N_{H}=4$ and $N_{T}=14$. The microstructures for different views are listed on the left and middle, and the radial density profiles are listed on the rightmost side. (**a1**) The front view of pure lipid molecule with $\phi_{\mathrm{NP}}=0$. (**a2**) The side view of pure lipid molecule. (**a3**) The radial density distribution of pure lipid molecule. (**b1**) The front view of lipid–nanoparticle mixtures with $\phi_{\mathrm{NP}}=0.03$. (**b2**) The side view of lipid–nanoparticle mixtures with $\phi_{\mathrm{NP}}=0.03$. (**b3**) The radial density distribution of lipid–nanoparticle mixtures with $\phi_{\mathrm{NP}}=0.03$. (**c1**) The front view of lipid–nanoparticle mixtures with $\phi_{\mathrm{NP}}=0.15$. (**c2**) The side view of lipid–nanoparticle mixtures with $\phi_{\mathrm{NP}}=0.15$. (**c3**) The radial density distribution of lipid–nanoparticle mixtures with $\phi_{\mathrm{NP}}=0.15$.

**Figure 5:**
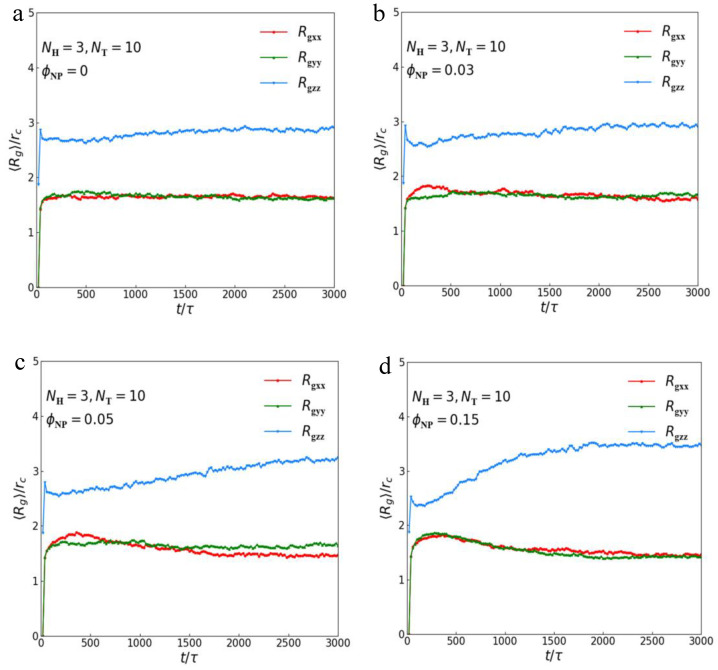
The average radius of gyration of lipid–nanoparticle mixtures with lamellar structure. There are three components $\langle R_{\mathrm{gxx}}\rangle$, $\langle R_{\mathrm{gyy}}\rangle$ and $\langle R_{\mathrm{gzz}}\rangle$ of $\langle R_{g}\rangle$ at different concentrations of nanoparticles as functions of time steps with (**a**) $\phi_{\mathrm{NP}}=0$, (**b**) $\phi_{\mathrm{NP}}=0.03$, (**c**) $\phi_{\mathrm{NP}}=0.05$ and (**d**) $\phi_{\mathrm{NP}}=0.15$.

**Figure 6:**
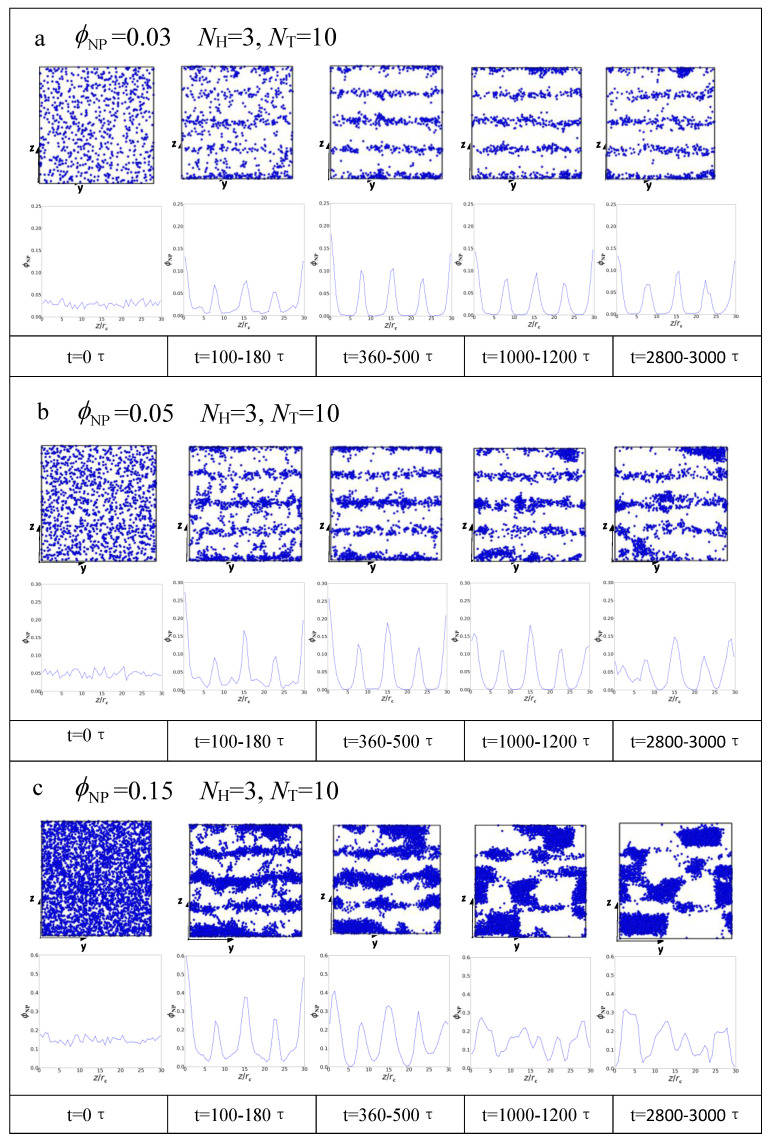
The dynamic processes for position distributions of nanoparticles in lamellar structures with (**a**) $\phi_{\mathrm{NP}}=0.03$, (**b**) $\phi_{\mathrm{NP}}=0.05$ and (**c**) $\phi_{\mathrm{NP}}=0.15$. The upper line shows the schematic diagram of nanoparticles at each time stage, and the lower line displays the corresponding density profiles of nanoparticles.

**Figure 7:**
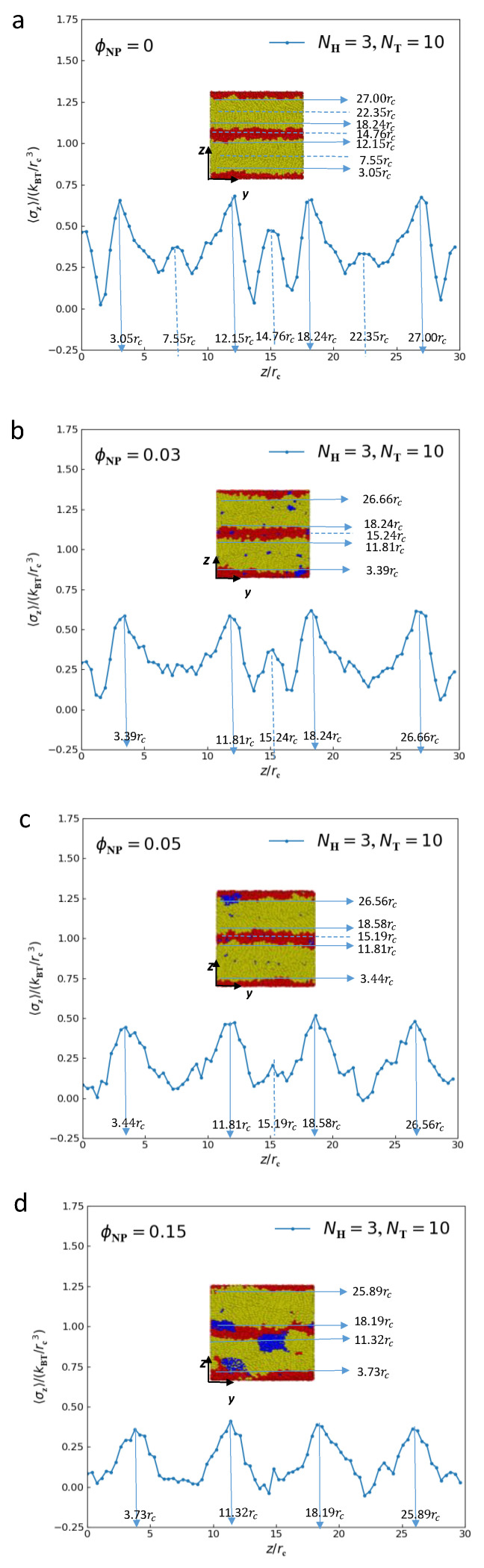
The average tensions ($\langle \sigma_{z}\rangle$) as a function of distance along the *z*-axis for lamellar structures with (**a**) $\phi_{\mathrm{NP}}=0$, (**b**) $\phi_{\mathrm{NP}}=0.03$, (**c**) $\phi_{\mathrm{NP}}=0.05$ and (**d**) $\phi_{\mathrm{NP}}=0.15$.

**Figure 8:**
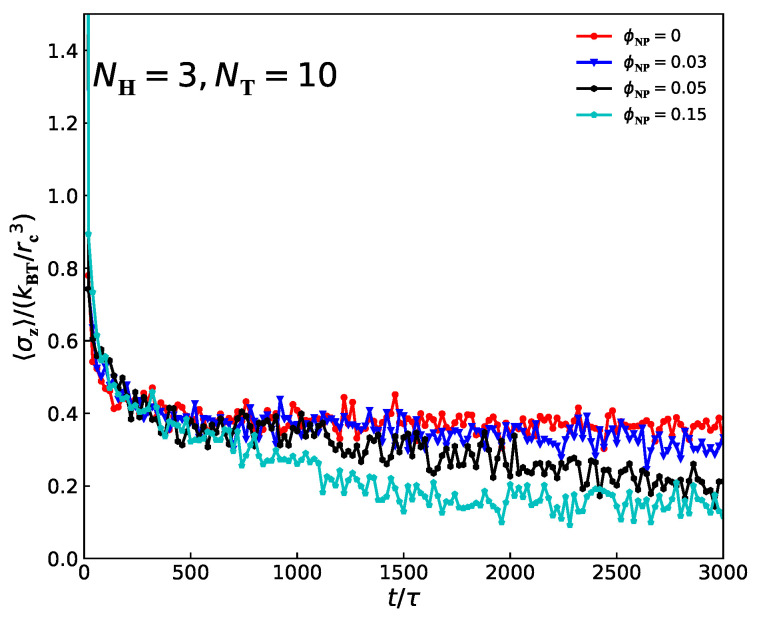
The average tensions ($\langle \sigma_{z}\rangle$) along the *z*-direction as a function of time for lamellar structures with $\phi_{\mathrm{NP}}=0$, $\phi_{\mathrm{NP}}=0.03$, $\phi_{\mathrm{NP}}=0.05$ and $\phi_{\mathrm{NP}}=0.15$.

In this section, we analyze and discuss the results from the DPD simulation for the self-assembly of pure lipid molecules and lipid–nanoparticle composites in a melt. In order to systematically study the influences of nanoparticles on the lipid matrix, different nanoparticle concentrations were set, including $\phi_{NP}=$ 0.03, 0.05 and 0.15, to represent low concentration, medium concentration and high concentration, respectively. The nanoparticles selected in this study are neutral, which indicates the absence of preferable interaction between nanoparticles and hydrophobic or hydrophilic parts of lipid molecules. The equilibrium structures are displayed in Figure 3 and Figure 4, the dynamic processes are demonstrated in Figure 5 and Figure 6 and the mechanical properties are illustrated in Figure 7 and Figure 8.

### 3.1. Equilibrium Structures

In general, the equilibrium structure of lipid–nanoparticle composites depends on several factors, such as the different block ratios of lipid molecules, that is, the number of head particles and tail particles of lipids ($N_{H},N_{T}$), the interaction between nanoparticles and lipid molecules ($a_{ij}$), the concentration of nanoparticles ($\phi_{NP}$), etc. This is similar to diblock copolymer–nanoparticle composites, which have been proven experimentally and theoretically [2,5].

First, we perform the DPD simulation on the phase separation of lipid molecules in a pure melt with $N_{H}=3$ and $N_{T}=10$, which shows the lamellar microphase-separated morphology (Figure 3a1,a2). These lamellae have multilayer structures because of the amphiphilicity of lipid molecules in bulk. Figure 3a3 demonstrates the density profiles of the head particles and tail particles of lipids along the z-direction. Several peaks are found in the density distribution: the red curve corresponds to the head particles, and the yellow curve corresponds to the tail particles. The *z*-coordinate positions corresponding to the peaks in these curves coincide with the centers of the microdomain of head and tail particles (Figure 3a1). This density distribution diagram exhibits the lamellar structure of pure molten lipid molecules.

The microstructure can only display the spatial distribution but cannot provide orientation properties. In quantitatively monitoring the orientational ordering of the lipid chains, we introduce the following order parameter [53,54]:

(10) $$\langle P\left(\cos\theta \right)\rangle =\langle \frac{3}{2}\cos^{2}\theta -\frac{1}{2}\rangle ,$$

where θ is the angle between the chain direction and the *z*-axis, and the bracket represents an ensemble average. When the chain direction is parallel to the *z* direction, the order parameter takes a value of 1. When the chain direction is perpendicular to the *z* direction, the order parameter takes a value of −0.5, whereas a value of 0 represents a complete disorder in the distribution [17]. Figure 3a4 shows that the order parameter of lipid chains is about 0.6 at the positions where the head particles are concentrated, which indicates that the lipid chains are nearly parallel to the *z* direction at these places because the rigidity of the head chains in the lipids in our study is greater than that of the tail chains. This phenomenon indicates that the lamellar structure has a liquid–crystalline characteristic with well-orientational orders.

Then, we randomly add nanoparticles into the pure melt to study the effects of nanoparticles on the lamellar phase separation of lipid molecules. The equilibrium self-assembled structures; density profiles of the microphase separation; and distributions of nanoparticles with $\phi_{\mathrm{NP}}=0.03$, $\phi_{\mathrm{NP}}=0.05$ and $\phi_{\mathrm{NP}}=0.15$ are shown in Figure 3b–d, respectively. Here, the concentration of nanoparticles ($\phi_{\mathrm{NP}}$) is defined as follows:

(11) $$\phi_{NP}=\frac{N_{\mathrm{NP}}}{D^{3}},$$

where $N_{\mathrm{NP}}$ represents the number of nanoparticles, and $D^{3}$ is the volume of the simulation box. As shown in Figure 3b1,b2, these snapshots of equilibrium self-assembled structures indicate that the nanoparticles tend to concentrate at the center of the microdomains, forming nanosheets within the lipid matrix, and the lamellar structure of the matrix can be preserved well when $\phi_{\mathrm{NP}}=0.03$. The density profile of the nanoparticles, which is magnified by a factor of 10 to make it more obvious, displays several peaks at the center of head-particle domains and tail-particle domains (Figure 3b3). Meanwhile, we observed a significant ‘crater’ in the center of the density profile of the hydrophilic head particles (Figure 3b3), indicating an exclusion of head particles from this region, which shows that the nanoparticles are localized within this cavity. The entire system is organized into a well-ordered ‘core–shell’ structure. These results are consistent with previous studies by Thompson et al. on diblock–nanoparticle mixtures to predict ordered phases based on mean field theory [24]. The phenomenon of $\phi_{\mathrm{NP}}=0.05$ is similar to that of $\phi_{\mathrm{NP}}=0.03$, as seen from Figure 3c. However, when the concentration of nanoparticles increases to $\phi_{\mathrm{NP}}=0.15$, the nanoparticles tend to form clusters (Figure 3d1,d2), and we can seen from Figure 3d3 that the layered distribution of nanoparticles significantly weakens where the peaks in the density distribution of nanoparticles are weaker than that shown in Figure 3b3,c3. However, the order parameters (Figure 3b4–d4) show that the orientational order of lipid molecules is almost not affected by the nanoparticles, even at $\phi_{\mathrm{NP}}$ of 0.15.

In the case of hexagonal lipid–nanoparticle composites with parameters $N_{H}=4$ and $N_{T}=14$ (Figure 4), we can observe that the head particles form cylindrical cores, and the tail particles are gathered around the column boundaries. The lipid molecules can self-assemble into a hexagonal structure in the bulk, which is consistent with the phase diagram of glycerol mono-oleate [16,20]. Figure 4a1,a2 display the front and side views of the hexagonal structure of a pure lipid molecule. The red and yellow curves in Figure 4a3 show the radial density profiles of head and tail particles, respectively. In Figure 4a3, the red curve reaches the peak value at the interface between two microdomains and then decreases to zero. Meanwhile, the yellow curve begins to increase gradually at the interface until it reaches the boundaries of other cylinders. This density distribution diagram is consistent with the hexagonal structure. When randomly distributed nanoparticles are loaded, the nanoparticles prefer to drive to the surface of the head-particle cylinders, as shown in Figure 4b1,b2, where blue beads represent nanoparticles. In addition, the peak of the density profiles of nanoparticles, which is magnified by a factor of 20 (Figure 4b3), appears at the position where the head and tail chains join. When the concentration of nanoparticles increases to $\phi_{\mathrm{NP}}=0.15$, the distribution of nanoparticles is not very regular according to the snapshot of microstructure (Figure 4c1,c2); however, the density profile of nanoparticles, which is magnified by a factor of 10 (Figure 4c3), shows an obvious peak value at the position where the head and tail chains join. That is to say that the nanoparticles tend to be segregated at the interface between two microdomains in the equilibrium structure in a hexagonal structure. Previous works have reported that nanoparticles segregate to the interface when the interfacial tension between two different domains is sufficiently large and the particles are neutral in block copolymer–nanoparticle composites [55,56,57]. Here, we find that nanoparticles segregate to the interface between two microdomains in the hexagonal structure but localize at the center of microdomains in the lamellar structure. This finding indicates that the distribution of nanoparticles depends on the structure of the lipid matrix, which has important guiding significance for the production of new lipid nanomaterials.

### 3.2. Dynamic Processes

In this subsection, we concentrate on the dynamic processes of lipid–nanoparticle mixtures to understand their formation mechanism. We investigate the distinct dynamic processes of lipid–nanoparticle mixtures using different concentrations of nanoparticles by calculating the average radius of gyration and the distribution of nanoparticles with time steps. The physical quantity of the average radius of gyration $\langle R_{g}\rangle$ [58] is an important parameter to describe the polymer size. Thus, the three components of average radius of gyration ($\langle R_{\mathrm{gxx}}\rangle$, $\langle R_{\mathrm{gyy}}\rangle$ and $\langle R_{\mathrm{gzz}}\rangle$), as functions of time steps, can reflect dynamic information about chain size in the three axes. The radius of gyration tensor can be expressed as follows:

(12) $$R_{g}^{2}=\left(\begin{array}{ccc} R_{\mathrm{gxx}}^{2} & R_{\mathrm{gxy}}^{2} & R_{\mathrm{gxz}}^{2}\\ R_{\mathrm{gyx}}^{2} & R_{\mathrm{gyy}}^{2} & R_{\mathrm{gyz}}^{2}\\ R_{\mathrm{gzx}}^{2} & R_{\mathrm{gzy}}^{2} & R_{\mathrm{gzz}}^{2} \end{array}\right).$$

Therefore, the element $R_{g\alpha \beta }^{2}$ is presented as follows:

(13) $$\langle R_{g\alpha \beta }^{2}\rangle =\frac{1}{N}\sum_{i}\langle \left(r_{i,\alpha }-r_{c,\alpha }\right)\left(r_{i,\beta }-r_{c,\beta }\right)\rangle ,$$

where α,β∈{x,y,z}, $r_{i,\alpha }$ and $r_{c,\alpha }$ represent the α coordinate of the *i*-th bead and the center of mass, respectively, and *N* refers to the number of chains. Since we can obtain spatial positions of each bead at every time step from simulation, we can determine the center of mass of each chain and calculate the element of the average radius of gyration tensor ($\langle R_{\mathrm{gxx}}^{2}\rangle$, $\langle R_{\mathrm{gyy}}^{2}\rangle$ and $\langle R_{\mathrm{gzz}}^{2}\rangle$) according to Equation (13); then, $\langle R_{\mathrm{gxx}}\rangle$, $\langle R_{\mathrm{gyy}}\rangle$ and $\langle R_{\mathrm{gzz}}\rangle$ are the square root of $\langle R_{\mathrm{gxx}}^{2}\rangle$, $\langle R_{\mathrm{gyy}}^{2}\rangle$ and $\langle R_{\mathrm{gzz}}^{2}\rangle$, respectively.

Previous studies have shown that the effect of nanoparticles on the size of polymer matrix is complex and that it is related to the type, size and dispersion state of nanoparticles. Mackay et al. [59] found that the polystyrene chain expanded and increased with the increase in nanoparticle concentration by neutron scattering of polystyrene/polystyrene nanoparticles, whereas a polystyrene/silica nanoparticle experiment showed that the size of the polystyrene chain was not affected [60,61]. In the current simulation, we focus on the average radius of gyration of lipid–nanoparticle mixtures with lamellar structures, where the parameters are $N_{H}=3$ and $N_{T}=10$. The average radius of gyration for pure lipid molecules and lipid–nanoparticle mixtures with different nanoparticle concentrations are shown in Figure 5a–d. The three components ($\langle R_{\mathrm{gxx}}\rangle$, $\langle R_{\mathrm{gyy}}\rangle$ and $\langle R_{\mathrm{gzz}}\rangle$) of $\langle R_{g}\rangle$ are listed during the dynamic processes to clearly show the effect of nanoparticles on the microstructures. In pure lipid, we find that the three components converge to a stable value in sufficient time, as shown in Figure 5a. During the self-assembly period, the average values of $\langle R_{\mathrm{gxx}}\rangle$ and $\langle R_{\mathrm{gyy}}\rangle$ increase rapidly initially and then reach a stable value; we observe that the average values of $\langle R_{\mathrm{gxx}}\rangle$ and $\langle R_{\mathrm{gyy}}\rangle$ are basically the same during the whole simulation process, and the average values of $\langle R_{\mathrm{gzz}}\rangle$ are much larger than $\langle R_{\mathrm{gxx}}\rangle$ and $\langle R_{\mathrm{gyy}}\rangle$. These results indicate the isotropic conformation of lipid molecules in the *x*–*y* plane, and the chains are arranged along the z axis, which is consistent with the microstructural morphologies and the order parameter profiles displayed in Figure 3. When nanoparticles are loaded (Figure 5b–d), we find that the values of $\langle R_{\mathrm{gzz}}\rangle$ increase with the increase in nanoparticle concentration, while the values of $\langle R_{\mathrm{gxx}}\rangle$ and $\langle R_{\mathrm{gyy}}\rangle$ do not change obviously. Moreover, we calculated the mean and standard deviation values of these parameters in equilibrium states to compare them quantitatively, namely $\langle R_{\mathrm{gxx}}\rangle =1.65\pm 0.17r_{c}$, $\langle R_{\mathrm{gyy}}\rangle =1.62\pm 0.18r_{c}$ and $\langle R_{\mathrm{gzz}}\rangle =2.87\pm 0.07r_{c}$ for $\phi_{\mathrm{NP}}=0$; $\langle R_{\mathrm{gxx}}\rangle =1.60\pm 0.12r_{c}$, $\langle R_{\mathrm{gyy}}\rangle =1.64\pm 0.12r_{c}$ and $\langle R_{\mathrm{gzz}}\rangle =2.92\pm 0.13r_{c}$ for $\phi_{\mathrm{NP}}=0.03$; $\langle R_{\mathrm{gxx}}\rangle =1.46\pm 0.06r_{c}$, $\langle R_{\mathrm{gyy}}\rangle =1.59\pm 0.12r_{c}$ and $\langle R_{\mathrm{gzz}}\rangle =3.15\pm 0.16r_{c}$ for $\phi_{\mathrm{NP}}=0.05$; and $\langle R_{\mathrm{gxx}}\rangle =1.47\pm 0.12r_{c}$, $\langle R_{\mathrm{gyy}}\rangle =1.42\pm 0.11r_{c}$ and $\langle R_{\mathrm{gzz}}\rangle =3.47\pm 0.28r_{c}$ for $\phi_{\mathrm{NP}}=0.15$. These results indicate that the lipid chains are stretched in the z direction with the addition of nanoparticles.

We also investigate the dynamic processes of lamellar structures with different concentrations of nanoparticles by illustrating the distribution of nanoparticles with time steps. Figure 6 shows the variation in the distribution of nanoparticles with different concentrations of nanoparticles over time with $N_{H}=3$ and $N_{T}=10$. The upper part shows the distribution of nanoparticles at different time stages, and the lower part shows the corresponding density profiles of nanoparticles. Figure 6a illustrates the concentration of nanoparticles at $\phi_{\mathrm{NP}}=0.03$. We can observe that the nanoparticles are randomly distributed during the initial stage and then gradually present a layered distribution along the z axis with time. The appearance of several peaks corresponding to the center of microdomains on the corresponding density profiles of nanoparticles from t=100τ confirm this. When $\phi_{\mathrm{NP}}$ increases to 0.05, we find that the nanoparticles begin to form clusters from t=1000τ ( Figure 6b); however, the corresponding density profiles of nanoparticles still show layered distribution during the whole simulation process at this concentration. When $\phi_{\mathrm{NP}}$ reaches 0.15, as shown in Figure 6c, the nanoparticles are distributed from random to layered; then, from t=1000τ, a large number of nanoparticles form clusters, and the layered distribution of nanoparticles disappears, which can be seen from the corresponding density profiles of nanoparticles.

Because the nanoparticles are amphiphobic in our simulation, at low concentrations, the entropy of nanoparticles dominates; therefore, they tend to disperse in the system. However, owing to the close packing of rod-like lipid chains, the free volume is much greater in the central zone of each layer, providing more space for nanoparticles [62,63]. Localizing nanoparticles in these spaces sacrifices some translational entropy of the nanoparticles but avoids an even larger chain stretching penalty incurred by distributing the nanoparticles throughout the domain (Figure 3b and Figure 6a). When the nanoparticle concentration is increased, the distribution of nanoparticles around the center of domains narrows, and nanoparticles are densely packed around the center of the lipid domain. The nanoparticle dispersion within the domain becomes progressively more unfavorable as the lipid chains stretch farther to accommodate more particles. This increase in stretching penalty cannot be offset by nanoparticle translation entropy, thereby preventing nanoparticles from spreading throughout the domains. More nanoparticles are localized near the center of the lipid domain to avoid exceedingly large stretching penalty. Finally, above the threshold concentration of nanoparticles, the excess nanoparticles cannot assemble in the lipid domain, and the system presents an ordered lipid/nanoparticle phase coexisting with a macrophase separation of nanoparticles. As shown in Figure 3d and Figure 6c, for $\phi_{\mathrm{NP}}=0.15$, large clusters of nanoparticles form, and the lipid chains are stretched in the z direction. This behavior is similar to that reported in the experimental study conducted by Kim et al. in symmetric PS-b-P2VP block copolymers blended with gold nanoparticles [64].

### 3.3. Mechanical Properties

In this subsection, we present the mechanical properties of lipid–nanoparticle composites with different concentrations of nanoparticles by calculating the interfacial tensions. The interfacial tension of the lipid membrane has elicited considerable interest in recent years [65,66,67].

Based on the Irving–Kirkwood definition, the formulation of tension ($\sigma_{z}$) along the z direction is presented as follows [68,69,70,71,72]:

(14) $$\langle \sigma_{z}\rangle =\langle p_{zz}\rangle -\frac{1}{2}\left(\langle p_{xx}\rangle +\langle p_{yy}\rangle \right),$$

where the component of the pressure tensor ($P_{zz}$) can be achieved as follows:

(15) $$\langle p_{xx}\rangle =\frac{1}{V}\langle \sum_{i=1}^{N}m_{i}v_{ix}v_{ix}+\sum_{i=1}^{N}\sum_{j>i}^{N}F_{ijx}x_{ij}\rangle .$$

where *V* and *N* are the volume and the number of DPD beads in the simulated box, respectively; $x_{ij}$ and $F_{ijx}$ denote the relative position and force between *i*-th and *j*-th particles along the *x*-axis, respectively; and the components $P_{yy}$ and $P_{zz}$ have the same formula as $P_{xx}$, only with changes in the corresponding subscripts.

Figure 7 displays the interfacial tension ($\langle \sigma_{z}\rangle$) as a function of distance along the *z* direction for the lamellar structure of lipid–nanoparticle mixtures with different nanoparticle concentrations. In pure lipid molecules (Figure 7a), there several large peaks appear at positions of $z=3.05r_{c}$, 12.15$r_{c}$, 18.24$r_{c}$ and 27.0$r_{c}$. We find that these locations are almost located at the interfaces between microdomains, as shown in the illustration in Figure 7a. In addition, we also observe that there are several small peaks that appear at positions of $z=7.55r_{c}$, 14.76$r_{c}$ and 22.35$r_{c}$. These locations are the joins of two neighboring lipid chains arranged along the z direction. At positions away from these interfaces, the values of $\langle \sigma_{z}\rangle$ tend to be zero. The distributions of the interfacial tension ($\langle \sigma_{z}\rangle$) for $\phi_{\mathrm{NP}}=0.03$, $\phi_{\mathrm{NP}}=0.05$ and $\phi_{\mathrm{NP}}=0.15$ are similar to those of pure lipid molecules, except the values of large peaks and small peaks decrease with the increase in nanoparticle concentration. We can calculate that the average values of large peaks for pure molten lipid molecules, $\phi_{\mathrm{NP}}=0.03$, $\phi_{\mathrm{NP}}=0.05$ and $\phi_{\mathrm{NP}}=0.15$ are 0.654, 0.593, 0.501 and 0.390, respectively. The average values of small peaks for pure molten lipid molecules are about 0.390, and the small peaks disappear gradually when the nanoparticles are added, as shown in Figure 7b–d. This means the average interfacial tension ($\langle \sigma_{z}\rangle$) decreases with the increase in nanoparticles concentration. Moreover, we can determine the size of domain space based on the difference of the z-axis positions corresponding to these large peaks; the results are as follows: the size of domain spaces for tail beads are 9.1$r_{c}$, 8.42$r_{c}$, 8.37$r_{c}$ and 7.59$r_{c}$ for pure lipid molecules, $\phi_{\mathrm{NP}}=0.03$, $\phi_{\mathrm{NP}}=0.05$ and $\phi_{\mathrm{NP}}=0.15$, respectively, and the size of domain spaces for head beads are 6.09$r_{c}$, 6.43$r_{c}$, 6.70$r_{c}$ and 6.87$r_{c}$, respectively. That is to say that the size of domain space for head beads increases with the addition of nanoparticles, while the size of domain space for tail beads decreases with the addition of nanoparticles. This phenomenon is reasonable because the rigid head chain of lipid move apart to provide enough space for nanoparticles, which makes their thickness increase, while the flexible tail chains free up space for nanoparticles by curling them, which causes a reduction in their thickness.

In addition, we investigate the interfacial tension ($\langle \sigma_{z}\rangle$) changes with time steps for the lamellar structures of pure molten lipid molecules and lipid–nanoparticle mixtures (Figure 8). The red curve corresponds to pure molten lipid molecules; the blue curves correspond to $\phi_{\mathrm{NP}}=0.03$; and the black and green curves correspond to $\phi_{\mathrm{NP}}=0.05$ and $\phi_{\mathrm{NP}}=0.15$, respectively. In these cases, tension evolution can be divided into three parts: first, between the time of 0 and 500τ, the tension decreases rapidly, which corresponds to the random generation stage. From 500 to 2000τ, the tension shows a weak declining trend, which corresponds to the adjustment perforation stage. At this stage, the structure is still undergoing minor adjustments and evolution. Finally, the value of interfacial tensions reaches a stable value, which means the structures have already reached an equilibrium state. In addition, after reaching equilibrium states, the mean values of internal tensions are 0.36, 0.31, 0.23 and 0.15 for pure lipid molecules, $\phi_{\mathrm{NP}}=0.03$, $\phi_{\mathrm{NP}}=0.05$ and $\phi_{\mathrm{NP}}=0.15$, respectively. This means that the addition of nanoparticles seems to remove the interfacial tension of lipid molecules. This phenomenon is consistent with the experimental results reported by Chung et al., who showed that the introduction of silica nanoparticles into a binary polymer system can prevent the phase separation of the two polymers by gathering the nanoparticles at the interfaces [73].

## 4. Summary

In this work, the self-assembly behavior of lipid–nanoparticle mixtures are simulated by the DPD method. Two common structures, that is, lamellar and hexagonal structures, are observed in equilibrium states. By analyzing bead density distributions and order parameters of the equilibrium structure, we observe that nanoparticles tend to concentrate at the center of microdomains, forming nanosheets within the lipid matrix at a low nanoparticle concentration and forming clusters with the increase in nanoparticle concentration in lamellar structures. In contrast, in hexagonal structures, we find that nanoparticles segregate to the interface between two microdomains. This result indicates that the distribution of nanoparticles depends on the structure of the lipid matrix, which has important guiding significance for the production of new nanomaterials.

Then, we investigate the dynamic process of lamellar structures by calculating the average radius of gyration and the distribution of nanoparticles with time steps. Three components ($\langle R_{\mathrm{gxx}}\rangle$, $\langle R_{\mathrm{gyy}}\rangle$ and $\langle R_{\mathrm{gzz}}\rangle$) of $\langle R_{g}\rangle$ are listed during the dynamic process to clearly show the effect of nanoparticles on the microstructure of the lipid matrix. The average values of $\langle R_{\mathrm{gzz}}\rangle$ are much larger than $\langle R_{\mathrm{gxx}}\rangle$ and $\langle R_{\mathrm{gyy}}\rangle$, and the average values of $\langle R_{\mathrm{gxx}}\rangle$ and $\langle R_{\mathrm{gyy}}\rangle$ are almost the same, indicating the isotropic conformation of lipid molecules in the *x*–*y* plane and that the chains are arranged along the z axis. The increase in $\langle R_{\mathrm{gzz}}\rangle$ with the increase in nanoparticle concentration indicates that the lipid chains are stretched in the z direction with the addition of nanoparticles.

We also investigate the mechanical properties of lipid–nanoparticle composites with different concentrations of nanoparticles by calculating the interfacial tensions. We observe that there several large peaks appear at positions of the interfaces between microdomains, and several small peaks appear in the middle of microdomains where the two neighboring lipid chains arranged along the z direction join. By determining the z axis of these large peaks, we can calculate the size of the microdomain space, and we find that the size of the domain space for head beads increases with the addition of nanoparticles, while the size of the domain space for tail beads decrease with the addition of nanoparticles. In addition, the average interfacial tension decreases with the increase in nanoparticle concentration after reaching equilibrium states. That is to say that the addition of nanoparticles can remove the interfacial tension between lipids molecules.

## Figures and Tables

**Table 1:** The interaction parameter introduced in this article. H and T represent head and tail beads of lipid chain, N denotes nanoparticle beads.

$a_{\mathrm{ij}}$	**H** 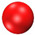	**T** 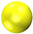	**N** 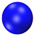
**H** 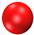	**25**		
**T** 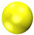	**100**	**25**	
**N** 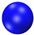	**100**	**100**	**25**

## Data Availability

The data presented in this study are available in the article.
